# Type 2 Diabetes and Glycemic Traits Are Not Causal Factors of Osteoarthritis: A Two-Sample Mendelian Randomization Analysis

**DOI:** 10.3389/fgene.2020.597876

**Published:** 2021-01-13

**Authors:** Zhiyong Cui, Hui Feng, Baichuan He, Yong Xing, Zhaorui Liu, Yun Tian

**Affiliations:** ^1^Department of Orthopedic Surgery, Peking University Third Hospital, Beijing, China; ^2^Peking University Health Science Center, Beijing, China; ^3^Peking University Sixth Hospital, Beijing, China

**Keywords:** Osteoarthritis, type 2 diabetes, fasting glucose, 2-h postprandial glucose, Mendelian randomization

## Abstract

**Background:**

It remains unclear whether an increased risk of type 2 diabetes (T2D) affects the risk of osteoarthritis (OA).

**Methods:**

Here, we used two-sample Mendelian randomization (MR) to obtain non-confounded estimates of the effect of T2D and glycemic traits on hip and knee OA. We identified single-nucleotide polymorphisms (SNPs) strongly associated with T2D, fasting glucose (FG), and 2-h postprandial glucose (2hGlu) from genome-wide association studies (GWAS). We used the MR inverse variance weighted (IVW), the MR–Egger method, the weighted median (WM), and the Robust Adjusted Profile Score (MR.RAPS) to reveal the associations of T2D, FG, and 2hGlu with hip and knee OA risks. Sensitivity analyses were also conducted to verify whether heterogeneity and pleiotropy can bias the MR results.

**Results:**

We did not find statistically significant causal effects of genetically increased T2D risk, FG, and 2hGlu on hip and knee OA (e.g., T2D and hip OA, MR–Egger OR = 1.1708, 95% CI 0.9469–1.4476, *p* = 0.1547). It was confirmed that horizontal pleiotropy was unlikely to bias the causality (e.g., T2D and hip OA, MR–Egger, intercept = −0.0105, *p* = 0.1367). No evidence of heterogeneity was found between the genetic variants (e.g., T2D and hip OA, MR–Egger *Q* = 30.1362, *I*^2^ < 0.0001, *p* = 0.6104).

**Conclusion:**

Our MR study did not support causal effects of a genetically increased T2D risk, FG, and 2hGlu on hip and knee OA risk.

## Introduction

Osteoarthritis (OA) and type 2 diabetes (T2D) are two pandemic chronic diseases and have significant impact on quality of life, social expenditure, and life expectancy (Martel-Pelletier et al., 2016; Hunter and Bierma-Zeinstra, 2019). OA is the most common chronic joint disease, and its main characteristic is the loss of chronic irreversible articular cartilage (Martel-Pelletier et al., 2016; Hunter and Bierma-Zeinstra, 2019). T2D is one category of diabetes, which is a chronic metabolic syndrome characterized by increased blood glucose levels as a consequence of insulin resistance (Chatterjee et al., 2017). The epidemiological association between T2D and OA was confirmed by different analyses showing that the prevalence of OA is significantly higher among patients with T2D than among those without the condition, and vice versa (Cheng et al., 2012; Alenazi et al., 2019). Cheng et al. (2012) found that the unadjusted prevalence of arthritis was 48.1% among United States adults with diabetes. Alenazi et al. (2019) also reported that the overall prevalence of T2D among patients with OA was about 16.6%. OA and T2D share common risk factors, such as obesity, physical activity, and genetic factors (Martel-Pelletier et al., 2016; Reyes et al., 2016; Chatterjee et al., 2017), which could explain why there was increasing prevalence of OA in T2D. However, the strength of such an association may have been heterogeneous by considering the heterogeneity of age, sex, ethnic group, duration of T2D, and body weight (Cannata et al., 2020). Since the exact mechanism involving T2D and OA was still debatable, it was controversial whether there was a causal relationship between T2D and OA. Recognizing the causal associations between the two diseases would have clinical implications for diseases management and be of great value for the design of specific therapeutic interventions targeting T2D and OA main pathogenic hallmarks.

Observational studies to estimate the causal inference have numerous inherent limitations, such as remaining limited to known and properly measured confounders (Greenland and Morgenstern, 2001). Therefore, we used Mendelian randomization (MR), an application of the method of instrumental variables (IVs), to the analysis of genetic data to assess the causal associations of T2D and related glycemic traits [fasting glucose (FG) and 2-h postprandial glucose (2hGlu)] with hip and knee OA. The genetic variants in MR are available with the progress of genome-wide association studies (GWAS) and high-throughput genomic technologies. MR method aims to give estimates of the causal effect free from biases due to confounding. Confounding factors are mitigated due to random assortment of genetic variants during meiosis yielding a random distribution of genetic variants in a population. MR method can also prevent reverse causality because genetic variants are fixed at conception and cannot be affected by disease processes. Moreover, the causal associations tested based on the MR method likely reflect the longstanding effect of exposures on outcomes for the genetic instruments that generally represent lifelong exposures (Emdin et al., 2017). In this study, we used single-nucleotide polymorphisms (SNPs) strongly associated with T2D and glycemic traits as IVs. We performed a two-sample MR and used statistical methods to obtain quantitative estimates to investigate the effect of T2D, FG, and 2hGlu upon hip and knee OA.

## Materials and Methods

### SNP Selection

In our study, we selected SNPs as IVs for all exposures (T2D, FG, and 2hGlu) and outcomes (hip and knee OA) from the IEU GWAS database, a database of genetic associations from GWAS summary datasets^1^ (Hemani et al., 2018). When target SNPs were not available in the outcome study, we used proxy SNPs that were in high linkage disequilibrium (LD) (*r*^2^ > 0.8) with the SNPs of interest. We selected the reference sample formed by the European ancestral individuals from the 1000 genomes project to estimate the allele frequency and LD level^2^ (1000 Genomes Project Consortium et al., 2010). The palindromic SNPs with intermediate allele frequencies (palindromic SNPs referred to the SNPs with A/T or G/C alleles and “intermediate allele frequencies” referred to 0.01 < allele frequency < 0.30) were excluded from the above selected instrument SNPs. SNPs with a minor allele frequency (MAF) of < 0.01 were also excluded. We also calculated the F statistics for the SNPs to measure the strength of the instruments. IVs with an F statistic less than 10 were excluded and were often labeled as “weak instruments” (Burgess et al., 2015). These rigorously selected SNPs were used as the final instrumental SNPs for the subsequent MR analysis.

### Data Source

Single-nucleotide polymorphisms associated with T2D were derived from a meta-analysis of GWAS in a very large sample of T2D (62,892 cases and 596,424 controls) of European ancestry (Xue et al., 2018). SNPs associated with FG and 2hGlu were derived from genome-wide association meta-analyses of up to 133,010 and 42,854 individuals, respectively, with males and females, of European ancestry without diabetes, performed by the Meta-Analyses of Glucose and Insulin-related traits Consortium (MAGIC) (Scott et al., 2012). The summary-level data for the impact of the exposures-associated SNPs on hip and knee OA were extracted from a genome-wide meta-analysis for OA of European descent with the UK Biobank and Arthritis Research UK Osteoarthritis Genetics (arcOGEN) resources (Tachmazidou et al., 2019). The self-reported OA status established during interview with a nurse and the Hospital Episode Statistics International Classification of Diseases, 10th edition (ICD 10) primary and secondary codes were used in the UK Biobank cases (Tachmazidou et al., 2019). The arcOGEN case samples were collected on the basis of clinical evidence of disease to a level requiring joint replacement or radiographic evidence of disease (Kellgren–Lawrence grade ≥ 2) (Tachmazidou et al., 2019). The detailed characteristics of GWAS associated with exposures (T2D, FG, and 2hGlu) and outcomes (hip and knee OA) are shown in Supplementary Table 1 in the Supplementary Material.

### Effect Size Estimate

We applied the two-sample MR to assess the role of exposures (T2D, FG, and 2hGlu) in the susceptibility of outcomes (hip and knee OA) (Hemani et al., 2018). We assessed the independent association of SNPs with T2D, FG, and 2hGlu and selected SNPs that were strongly associated (*p* < 5E-08) and independent inheritance (*r*^2^ < 0.01) without any LD with the exposures. Then, we obtained the effect estimates for the selected SNPs on hip and knee OA from genome-wide meta-analysis for OA in 2019. The causal associations between exposures (T2D, FG, and 2hGlu) and outcomes (hip and knee OA) were estimated with inverse variance weighted (IVW), MR–Egger, and weighted median (WM) (Burgess et al., 2013; Bowden et al., 2015). The IVW method uses a meta-analysis approach to combine the Wald ratios of the causal effects of each SNP and can provide the most precise estimates. However, it can be influenced by invalid IVs and pleiotropic effects. The WM estimate provides a reliable effect estimate of the causal effect when at least 50% of the weight in the analysis comes from effective IVs. MR–Egger regression is based on the assumption that the pleiotropic associations are independent, performs a weighted linear regression of the outcome coefficients on the exposure coefficients. MR–Egger estimates may be inaccurate and can be strongly influenced by outlying genetic variants (Bowden et al., 2016). We also performed a recently developed method called the Robust Adjusted Profile Score (MR.RAPS) to estimate the causal effects, which can lead to a considerably higher statistical power than the conventional MR analysis can, which only uses a small set of strong instruments (Zhao et al., 2019). MR.RAPS considers the measurement error in SNP-exposure effects, is unbiased when there are many weak instruments, and is robust to systematic and idiosyncratic pleiotropy (Zhao et al., 2019).

### Sensitivity Analyses

We used the IVW, WM, and maximum likelihood methods to evaluate the heterogeneity between SNPs. The heterogeneity was quantified by Cochran *Q* statistics and *I*^2^ statistics (Bowden et al., 2016). Moreover, we used the MR–Steiger method to compute the amount of variance instrumenting SNPs explained in the exposure and outcome variable and test if the variance in the outcome is less than the exposure. In case of a true causal direction, SNPs should be more predictive of the exposure than of the outcome (Hemani et al., 2017). Pleiotropy refers to the phenomenon in which a single locus affects multiple phenotypes. Horizontal pleiotropy arises when a genetic variant associates with more than one phenotype on separate pathways, which can invalidate the results from MR analyses (Stearns, 2010; Larsson et al., 2019). In order to explore and adjust for horizontal pleiotropy, we evaluated the pleiotropic effects of T2D and glycemic traits on weight-associated factors, including body mass index (BMI), weight, and obesity, as these confounding effects might distort the effects of T2D and glycemic traits on OA. Summary statistics for BMI were extracted from studies performed by the Genetic Investigation of ANthropometric Traits (GIANT) consortium (Locke et al., 2015), weight (male and female) from GIANT Consortium (Randall et al., 2013), and obesity from GIANT Consortium (Berndt et al., 2013). The detailed characteristics of studies associated with confounding factors are shown in Supplementary Table 1. We assessed the potential associations between SNPs that were extracted for the MR analysis and those confounding factors. Associations of the SNPs with the four confounding factors were considered statistically significant at a Bonferroni-corrected *p* < 0.05/(4 × *N*), with *N* representing the number of SNPs in each exposure trait. In addition to evaluating the associations with the risk factors, we performed MR–Egger regression to explore and adjust for horizontal pleiotropy, which was a method that can provide evidence for confounders that would distort the MR results. The intercept represents the average pleiotropic effect across the genetic variants.

### Power Assessment

We also used an online tool mRnd^3^ to calculate the power to detect causal effect. The equations using an approximate linear model on the observed binary (0–1) scale were adapted for binary outcomes, which needs several parameters to estimate. These parameters include the proportion of phenotypic variation explained by IV SNPs, the effect size of the exposure to the outcome at the epidemiological level, sample size, and standard deviation of exposure and outcome (Brion et al., 2013).

The results of the MR analyses were considered statistically significant at a Bonferroni-corrected *p* < 0.025 (e.g., 0.05/2 outcomes). All statistical tests were two-sided and performed using the “TwoSampleMR” package for R language, version 3.6.1 (R Foundation for Statistical Computing, Vienna, Austria).

## Results

### Causality Between T2D and OA

For T2D, we used 37 genome-wide significant (*p* < 5E-08) SNPs associated with increased T2D risk identified in the largest meta-analysis of T2D GWAS (Xue et al., 2018). For each of the susceptibility variants for T2D, we sought summary-level data for OA from the GWAS performed by arcOGEN Consortium. After removing two T2D variants (rs6494307, rs7619041) that were palindromic with intermediate allele frequencies, 35 SNPs remained to perform the MR analysis for hip and knee OA. None of the proxy SNPs were used in the analysis. For these IVs, all the *F* values were larger than 10, ranging from 30.6746 to 256.3266, with an average *F* value of 53.4983 (Supplementary Table 2).

**FIGURE 1:**
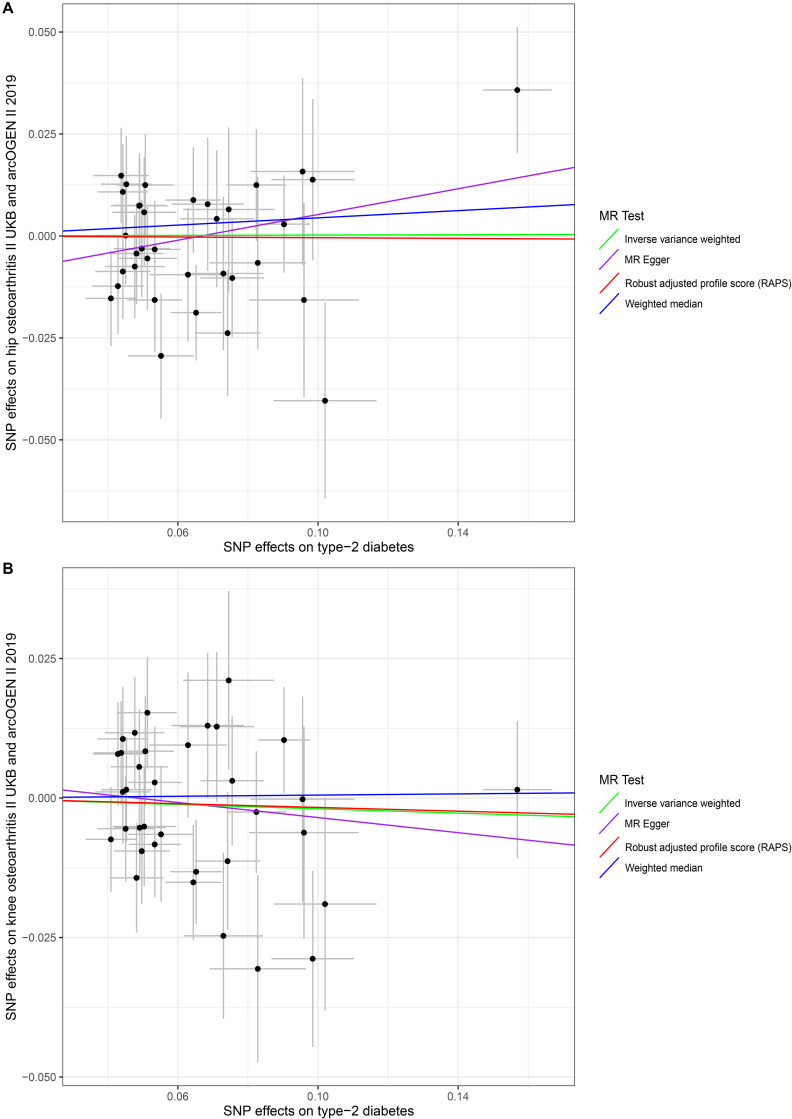
Scatter plots of genetic associations with type 2 diabetes risk against osteoarthritis using different MR methods. **(A)** Type 2 diabetes and hip osteoarthritis results; **(B)** type 2 diabetes and knee osteoarthritis results. The slopes of each line represent the causal association for each method. The green line represents the inverse variance weighted estimate, the purple line represents the MR–Egger estimate, the red line represents the MR.RAPS estimate, and the blue line represents the weighted median estimate.

In our analysis using the full set of 35 SNPs, we did not find causal associations of per unit increase in the log-odds of having T2D with risk changes of having OA, based on the IVW, WM, MR–Egger regression, and MR.RAPS methods at the Bonferroni-corrected significance threshold *p* < 0.025 (e.g., 0.05/2). (For hip OA, MR–Egger OR = 1.1708, 95% CI 0.9469–1.4476, *p* = 0.1547; IVW OR = 1.0022, 95% CI 0.9329–1.0767, *p* = 0.9517; WM OR = 1.0454, 95% CI 0.9369–1.1664, *p* = 0.4274; MR.RAPS OR = 0.9957, 95% CI 0.9239–1.0731, *p* = 0.9094. For knee OA, MR–Egger OR = 0.9046, 95% CI 0.7880–1.1085, *p* = 0.4426; IVW OR = 0.9809, 95% CI 0.9265–1.0385, *p* = 0.5084; WM OR = 1.0053, 95% CI 0.9213–1.0971, *p* = 0.9050; MR.RAPS OR = 0.9833, 95% CI 0.9247–1.0457, *p* = 0.5920.) (Table 1 and Figure 1). We assessed the horizontal pleiotropy by checking the association of T2D-associated SNPs with confounders, and no significant association signal was detected among the 35 SNPs we selected at the Bonferroni-corrected significance threshold *p* < 3.57E-04 (e.g., 0.05/140) (Supplementary Table 3). We also assessed the horizontal pleiotropy with the MR–Egger regression and found that no horizontal pleiotropy would bias the causality with hip OA (intercept = −0.0105, *p* = 0.1367) and knee OA (intercept = 0.0033, *p* = 0.5589) (Table 2). The heterogeneity test demonstrated that there is no evidence of heterogeneity in the MR analysis. For hip OA, MR–Egger *Q* = 30.1362, *I*^2^ < 0.0001, *p* = 0.6104; IVW *Q* = 32.4634, *I*^2^ < 0.0001, *p* = 0.5430; maximum likelihood *Q* = 32.4633, *I*^2^ < 0.0001, *p* = 0.5430. For knee OA, MR–Egger *Q* = 33.5130, *I*^2^ = 0.0153, *p* = 0.4424; IVW *Q* = 33.8671, *I*^2^ < 0.0001, *p* = 0.4741; maximum likelihood *Q* = 33.8592, *I*^2^ < 0.0001, *p* = 0.4745. (Table 2). The MR–Steiger results supported that the SNPs in the analysis were more predictive of the exposure than of the outcome (Table 1). Power calculations showed that our sample provided sufficient statistical power (>80%) for causal analysis of T2D on hip and knee OA (Table 3).

**TABLE 1:** MR estimates from each method of assessing the causal effects of T2D, FG and 2hGlu on OA risk.

Exposure traits	MR methods	Hip OA					Knee OA				
		Number of SNPs	OR (95% CI)	SE	MR *p*-value	MR-Steiger test	Number of SNPs	OR (95% CI)	SE	MR *p*–value	MR-Steiger test
T2D	MR-Egger	35	1.1708 (0.9469∼1.4476)	0.1083	0.1547	Direction: TRUE *p*-value < 0.0001	35	0.9046 (0.7880∼1.1085)	0.0871	0.4426	Direction: TRUE *p*-value < 0.0001
	Inverse variance weighted	35	1.0022 (0.9329∼1.0767)	0.0366	0.9517		35	0.9809 (0.9265∼1.0385)	0.0291	0.5084	
	Weighted median	35	1.0454 (0.9369∼1.1664)	0.0559	0.4274		35	1.0053 (0.9213∼1.0971)	0.0446	0.9050	
	Robust adjusted profile score	35	0.9957 (0.9239∼1.0731)	0.0382	0.9094		35	0.9833 (0.9247∼1.0457)	0.0314	0.5920	
FG	MR-Egger	10	0.4634 (0.1848∼1.1617)	0.4690	0.1396	Direction: TRUE *p*-value < 0.0001	10	0.5890 (0.3697∼1.0943)	0.1665	0.1559	Direction: TRUE *p*-value < 0.0001
	Inverse variance weighted	10	0.9820 (0.6545∼1.4734)	0.2070	0.9301		10	0.8158 (0.6009∼1.1077)	0.1560	0.1921	
	Weighted median	10	0.6670 (0.4176∼1.0651)	0.2388	0.0899		10	0.6491 (0.4060∼1.1897)	0.1721	0.3417	
	Robust adjusted profile score	10	0.8996 (0.5811∼1.3926)	0.2229	0.6350		10	1.0374 (0.4699∼2.2900)	0.4040	0.9299	
2hGlu	MR-Egger	3	1.3062 (0.2540∼2.8190)	0.6140	0.8309	Direction: TRUE *p*-value < 0.0001	3	1.3652 (0.7171∼2.5993)	0.3285	0.5171	Direction: TRUE *p*-value < 0.0001
	Inverse variance weighted	3	1.1976 (0.9437∼1.5199)	0.1216	0.1380		3	1.0594 (0.9067∼1.2378)	0.0794	0.4673	
	Weighted median	3	1.3301 (1.0348∼1.7098)	0.1281	*0.0260*		3	1.0786 (0.8998∼1.2930)	0.0925	0.4133	
	Robust adjusted profile score	3	1.2125 (0.9817∼1.4976)	0.1077	0.0737		3	1.0598 (0.9020∼1.2451)	0.0822	0.4804	

*MR, Mendelian randomization; SNP, single nucleotide polymorphism; OA, osteoarthritis; T2D, type 2 diabetes; FG, fasting glucose; 2hGlu, 2-h postprandial glucose; OR, odds ratio; CI, confidence interval; SE, standard error (the standard error is an estimate of the standard deviation (SD) of the coefficient).*

**TABLE 2:** Heterogeneity and pleiotropy analysis of T2D, FG and 2hGlu with hip and knee OA risk using different analytic methods.

Exposure traits	MR methods	Hip OA					Knee OA				
		Cochran Q statistic	*I*^2^	Heterogeneity *p*-value	MR-Egger		Cochran Q statistic	*I*^2^	Heterogeneity *p*-value	MR-Egger	
					Intercept	Intercept *p*-value				Intercept	Intercept *p*-value
T2D	MR-Egger	30.1362	<0.0001	0.6104	−0.0105	0.1367	33.5130	0.0153	0.4424	0.0033	0.5589
	Inverse variance weighted	32.4634	<0.0001	0.5430			33.8671	<0.0001	0.4741		
	Maximum likelihood	32.4633	<0.0001	0.5430			33.8592	<0.0001	0.4745		
FG	MR-Egger	11.6746	0.2291	0.1663	0.0243	0.1189	13.8053	0.4205	0.0870	−0.0078	0.5348
	Inverse variance weighted	16.1241	0.4418	0.0643			14.5310	0.3806	0.1047		
	Maximum likelihood	16.1240	0.4418	0.0643			14.5175	0.3801	0.1051		
2hGlu	MR-Egger	2.2054	0.5466	0.1375	0.0303	0.6639	0.0204	<0.0001	0.8863	−0.0222	0.5722
	Inverse variance weighted	2.9551	0.3232	0.2282			0.6533	<0.0001	0.7213		
	Maximum likelihood	2.8701	0.3032	0.2381			0.6503	<0.0001	0.7224		

*MR, Mendelian randomization; OA, osteoarthritis; T2D, type 2 diabetes; FG, fasting glucose; 2hGlu, 2-hour postprandial glucose.*

**TABLE 3:** Power calculation for the MR analysis in current study.

Exposure		Outcome			OR	Proportion of variance explained by the SNPs on exposure	Power
Trait	Sample size	Trait	Sample size	Proportion of cases			
T2D	659,316	Hip OA	382,833	0.0336	1.1708	0.0508	99%
T2D	659,316	Knee OA	391,904	0.0559	0.9046	0.0508	87%
FG	133,010	Hip OA	382,833	0.0336	0.4634	0.0029	91%
FG	133,010	Knee OA	391,904	0.0559	0.5890	0.0029	91%
2hGlu	42,854	Hip OA	382,833	0.0336	1.3062	0.0074	83%
2hGlu	42,854	Knee OA	391,904	0.0559	1.3652	0.0074	99%

*T2D, type 2 diabetes; FG, fasting glucose; 2hGlu, 2-h postprandial glucose; OA, osteoarthritis; OR, odds ratio; SNP, single nucleotide polymorphism.*

### Causality Between FG in Non-diabetic Individuals and OA

Based on independent and LD analyses, we selected 10 genome-wide significant (*p* < 5E-08) SNPs associated with FG in non-diabetic individuals to analyze the causality with hip and knee OA, and no palindromic SNPs were found. None of the proxy SNPs were used in the analysis. The *F* values of the 10 SNPs ranged from 32.6531 to 442.4495, with an average value of 124.7024 (Supplementary Table 4).

**FIGURE 2:**
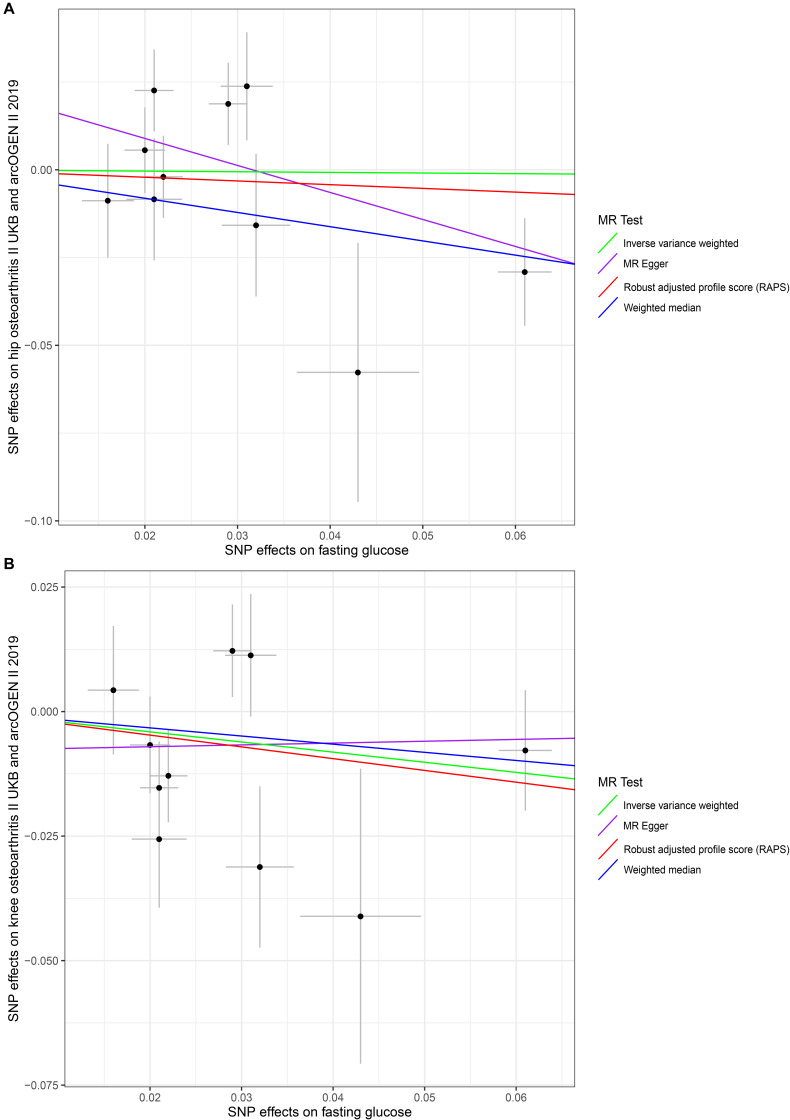
Scatter plots of genetic associations with fasting glucose against osteoarthritis using different MR methods. **(A)** Fasting glucose and hip osteoarthritis results; **(B)** fasting glucose and knee osteoarthritis results. The slopes of each line represent the causal association for each method. The green line represents the inverse variance weighted estimate, the purple line represents the MR–Egger estimate, the red line represents the MR.RAPS estimate, and the blue line represents the weighted median estimate.

No evidence supported that the genetically increased FG was causally associated with the hip and knee OA risk changes in non-diabetic individuals based on the IVW, WM, MR–Egger regression, and MR.RAPS methods (*p* < 0.025). For hip OA, MR–Egger OR = 0.4634, 95% CI 0.1848–1.1617, *p* = 0.1396; IVW OR = 0.9820, 95% CI 0.6545–1.4734, *p* = 0.9301; WM OR = 0.6670, 95% CI 0.4176–1.0651, *p* = 0.0899; MR.RAPS OR = 0.8996, 95% CI 0.5811–1.3926, *p* = 0.6350. For knee OA, MR–Egger OR = 0.5890, 95% CI 0.3697–1.0943, *p* = 0.1559; IVW OR = 0.8158, 95% CI 0.6009–1.1077, *p* = 0.1921; WM OR = 0.6491, 95% CI 0.4060–1.1897, *p* = 0.3417; MR.RAPS OR = 1.0374, 95% CI 0.4699–2.2900, *p* = 0.9299. (Table 1 and Figure 2). None of the 10 SNPs were significantly associated with known confounders at the Bonferroni-corrected significance threshold (*p* < 0.0013) (e.g., 0.05/40) (Supplementary Table 5). We also conducted the MR–Egger regression to assess the horizontal pleiotropy, and the results revealed that the horizontal pleiotropy was unlikely to bias the causality with hip OA (intercept = 0.0243, *p* = 0.1189) and knee OA (intercept = −0.0078, *p* = 0.5348) (Table 2).

We also found no significant heterogeneity between FG and OA. For hip OA, MR–Egger *Q* = 11.6746, *I*^2^ = 0.2291, *p* = 0.1663; IVW *Q* = 16.1241, *I*^2^ = 0.4418, *p* = 0.0643; maximum likelihood *Q* = 16.1240, *I*^2^ = 0.4418, *p* = 0.0643. For knee OA, MR–Egger *Q* = 13.8053, *I*^2^ = 0.4205, *p* = 0.0870; IVW *Q* = 14.5310, *I*^2^ = 0.3806, *p* = 0.1047; maximum likelihood *Q* = 14.5175, *I*^2^ = 0.3801, *p* = 0.1051. (Table 2). The MR–Steiger directionality test showed that the SNPs in the analysis were more predictive of the exposure than of the outcome (Table 1). We also have sufficient statistical power (>80%) to detect the true causal effect between FG and hip and knee OA (Table 3).

### Causality Between 2hGlu in Non-diabetic Individuals and OA

We chose three independent SNPs associated with 2hGlu in European ancestry from summary statistics datasets of GWAS meta-analyses, and no palindromic SNPs were found. The *F* values of the three SNPs were 32.8017, 33.5180, and 39.0625, with an average value of 35.1274 (Supplementary Table 6).

**FIGURE 3:**
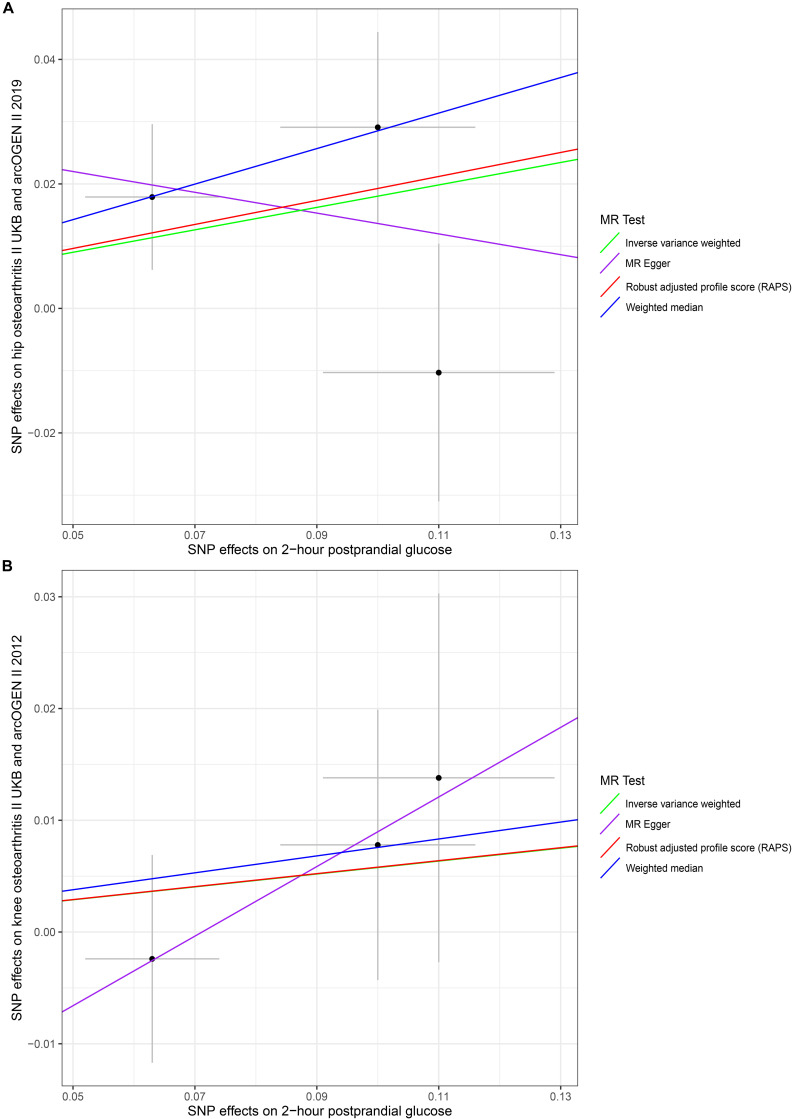
Scatter plots of genetic associations with 2-h postprandial glucose against osteoarthritis using different MR methods. **(A)** 2-h postprandial glucose and hip osteoarthritis results; **(B)** 2-h postprandial glucose and knee osteoarthritis results. The slopes of each line represent the causal association for each method. The green line represents the inverse variance weighted estimate, the purple line represents the MR–Egger estimate, the red line represents the MR.RAPS estimate, and the blue line represents the weighted median estimate.

Our results did not suggest causal associations of genetically increased 2hGlu with hip and knee OA risk changes in non-diabetic individuals (*p* < 0.025). (For hip OA, MR–Egger OR = 1.3062, 95% CI 0.2540–2.8190, *p* = 0.8309; IVW OR = 1.1976, 95% CI 0.9437–1.5199, *p* = 0.1380; WM OR = 1.3301, 95% CI 1.0348–1.7098, *p* = 0.0260; MR.RAPS OR = 1.2125, 95% CI 0.9817–1.4976, *p* = 0.0737. For knee OA, MR–Egger OR = 1.3652, 95% CI 0.7171–2.5993, *p* = 0.5171; IVW OR = 1.0594, 95% CI 0.9067–1.2378, *p* = 0.4673; WM OR = 1.0786, 95% CI 0.8998–1.2930, *p* = 0.4133; MR.RAPS OR = 1.0598, 95% CI 0.9020–1.2451, *p* = 0.4804.) (Table 1 and Figure 3). We conducted the MR–Egger regression to assess the pleiotropy, and the results revealed that the horizontal pleiotropy was unlikely to bias the causality with hip OA (intercept = 0.0303, *p* = 0.6639) and knee OA (intercept = −0.0222, *p* = 0.5722) (Table 1).

The associations between these genetic variants and confounding factors were analyzed. None of the three genetic variants were significantly associated with the confounding factors mentioned above at the Bonferroni-corrected significance threshold (*p* < 0.0042) (e.g., 0.05/12) (Supplementary Table 7). Cochran’s *Q* value and *I*^2^ value indicated no evidence of heterogeneity between IV estimates with the IVW, MR–Egger, and maximum likelihood methods. (For hip OA, MR–Egger *Q* = 2.2054, *I*^2^ = 0.5466, *p* = 0.1375; IVW *Q* = 2.9551, *I*^2^ = 0.3232, *p* = 0.2282; maximum likelihood *Q* = 2.8701, *I*^2^ = 0.3032, *p* = 0.2381. For knee OA, MR–Egger *Q* = 0.0204, *I*^2^ < 0.0001, *p* = 0.8863; IVW *Q* = 0.6533, *I*^2^ < 0.0001, *p* = 0.7213; maximum likelihood *Q* = 0.6503, *I*^2^ < 0.0001, *p* = 0.7224.) (Table 2). The MR–Steiger results supported that the SNPs in the analysis were more predictive of the exposure than of the outcome (Table 1). Power calculations showed that our sample provided sufficient statistical power (>80%) for causal analysis of 2hGlu on hip and knee OA (Table 3).

## Discussion

To our knowledge, this is the first MR study on the effect of T2D and other glycemic traits on OA. We obtained sufficient sample sizes and thus had sufficient power (>80%) to detect causal effects. We did not distinguish statistical causality between exposures and outcomes based on our MR results. The *F* values of IVs indicated that the variables satisfy the strong relevance assumption of MR, and that the instrument bias was weak and could not substantially influence the estimations of causal effects. We used the MR–Egger method to detect and adjust for pleiotropy of the genetic variants. We also performed heterogeneity and did not find significant heterogeneity between SNPs, which indicated the reliability of the MR results. Some studies reported similar results that there was no evidence to support the causal associations between T2D and OA. Frey et al. (2016) conducted one case–control study and provided evidence that T2D is not an independent risk factor for hand OA regardless of T2D severity, duration, or pharmacological treatment. Funck-Brentano et al. (2019) analyzed the individual-level data in UK Biobank study and performed MR analysis. They found no significant causality for T2D with all OA, knee OA, hip OA, and hand OA. Zengini et al. (2018) also found no causal association of T2D with self-reported OA or hospital-diagnosed OA with the MR analysis.

Some studies provided a few suggestive pathophysiological mechanisms for the development of OA in T2D patients. One of them was hyperglycemia-induced accelerated synthesis of Advanced Glycation End products (AGEs), which leads to an increase in oxidative stress. These AGEs have been regarded as one of the factors responsible for healing impairment and loss of elasticity of the cartilage (DeGroot et al., 2001). Another mechanism was that chronic high glucose environment had noxious effects on chondrocytes metabolism (Laiguillon et al., 2015). High glucose environment would induce diabetic cartilages to produce more interleukin-6 and prostaglandin E2. High glucose exposure also increased the metalloproteinases production especially in human OA chondrocytes and decreased the production of collagen II. Vaamonde-Garcia et al. (2017) demonstrated that high glucose environment favored the suppression of heme oxygenase-1, which led to an increase in the oxidative stress and cartilage damage. Chen et al. (2015) found that high glucose diminished the synthesis of type II collagen and peroxisome proliferator-activated receptor gamma (PPARγ) by chondrocytes, which could result in the development of cartilage defects.

Many studies reported the suggestive evidence to support the associations between T2D and OA. Eymard et al. (2015) demonstrated that T2D was a predictor of joint space reduction in men with established knee OA. Davies-Tuck et al. (2012) reported the evidence that increased FG concentration in non-diabetic individuals was associated with adverse structural changes at the knee in women based on one prospective cohort study. Williams et al. (2016) executed one meta-analysis including 10 observational studies with 16,742 patients in total. They revealed that T2D was associated with radiographic and symptomatic OA even after controlling the BMI and weight. Schett et al. (2013) reported that T2D could predict the progress of OA, independent of age and BMI, based on one cohort study followed up over 20 years. Although many studies suggested the associations, those evidences were too weak to indicate the causal associations between T2D and OA. Confounders might interfere with the associations between T2D and OA. Since T2D and OA share common risk factors, some studies even suggested that OA was the component of metabolic syndrome (Schett et al., 2013); metabolic factors, such as obesity, inflammatory factors, physical activity, and diabetic medication might have impacts on OA. For example, obesity is a pandemic condition defined as the abnormal or the excessive accumulation of fat, which is characteristic of T2D (Kolb and Martin, 2017). Obesity would promote the progress of OA through mechanical load and inflammatory reaction. Mechanical load means that the increase load of weight-bearing joint caused by obesity could accelerate the development of OA. Inflammatory reaction indicated that the increased systemic and local inflammation caused by obesity would damage the integrity of the extracellular matrix of the cartilage (Martel-Pelletier et al., 2016; Reyes et al., 2016). Furthermore, some confounders that cannot be entirely ruled out, such as socioeconomic status, occupation, and nutrition, also had impacts on the association between T2D and OA (Frey et al., 2016). Some studies (Schett et al., 2013) used joint arthroplasty due to OA as the study endpoint, which precludes the ability to assess temporality, because joint arthroplasty was the terminal event of OA. This would be the reason that affected the association between T2D and OA. Besides, reverse causation bias between T2D and OA (Kendzerska et al., 2018) would also limit the ability to provide causal estimates of the effect of exposures on outcomes in the observational studies.

The present study has several limitations. The criteria for OA were limited in the GWAS included in the study. The radiographic OA or the mild symptomatic OA was not included. Additionally, there were only two types of OA, hip and knee OA, involved in the study. The hand OA was not analyzed in the MR study, which might distinguish the pathogenesis mechanism from hip and knee OA due to the absence of weight-bearing factors. The samples included in the exposures and outcomes were of European ancestry, which could mitigate the population stratification. However, the conclusions based on the European sample were not representative of other ancestries, such as Asians and Americans. Moreover, we only evaluated the associations between SNPs and weight-associated confounders due to the limited publicly available GWAS databases. The associations between these instruments and other potential confounders, such as physical activity, were not evaluated in our study.

## Conclusion

In summary, our two-sample MR analysis did not suggest the significant causal effects of genetic increases in T2D risk, FG, and 2hGlu with hip and knee OA. The complicated effects of T2D risk, FG, and 2hGlu with OA might be influenced by other confounding factors, which still need further investigation in the future. In addition, future studies should additionally seek to investigate the effect of T2D and glycemic traits on hand OA.

## Data Availability Statement

The original contributions presented in the study are included in the article/Supplementary Material, further inquiries can be directed to the corresponding author.

## Author Contributions

YT, ZC, and BH conceptualized and designed the study. ZC and HF provided the “TwoSampleMR” package codes in R language and analyzed the data in the study. ZC drafted the manuscript. YX and ZL gave constructive suggestions when writing the manuscript. All authors have read the manuscript.

## Conflict of Interest

The authors declare that the research was conducted in the absence of any commercial or financial relationships that could be construed as a potential conflict of interest.

## Footnotes

1 https://gwas.mrcieu.ac.uk/

2 http://www.internationalgenome.org/

3 http://cnsgenomics.com/shiny/mRnd/
