# Supplementary material for: Type 2 Diabetes and Glycemic Traits Are Not Causal Factors of Osteoarthritis: A Two-Sample Mendelian Randomization Analysis
Source: Front Genet. 2021 Jan 13;11:597876. doi: 10.3389/fgene.2020.597876 (PMC7838644; doi:10.3389/fgene.2020.597876)
Supplement: Supplementary file 1 [file Data_Sheet_1.PDF]

Supplementary material

Table S1. The detailed characteristics of GWAS associated with exposures,outcomes and confounding factors.

Table S2. Characteristics of SNPs used in Mendelian randomization analysis of the effects of T2D on hip and knee OA risk.

Table S3. Associations of SNPs used in Mendelian randomization analysis of T2D and osteoarthritis risk with confounding factors.

Table S4. Characteristics of SNPs used in Mendelian randomization analysis of the effects of FG in nondiabetic individuals on hip and knee OA risk.

Table S5. Associations of SNPs used in Mendelian randomization analysis of FG and osteoarthritis risk with confounding factors.

Table S6. Characteristics of SNPs used in Mendelian randomization analysis of the effects of 2hGlu in nondiabetic individuals on hip and knee OA risk.

Table S7. Associations of SNPs used in Mendelian randomization analysis of 2hGlu and osteoarthritis risk with confounding factors.

Traits	Author	Year	Sample size	Data source	Population
T2D	Xue A et al (Xue et al. 2018)	2018	Genome-wide association meta-analysis with 62,892 T2D cases and 596,424 controls	DIAGRAM, GERA and UKB	European ancestry
FG and 2hGlu	Scott RA et al (Scott et al. 2012)	2012	Genome-wide association meta-analyses of 133,010 (FG), 108,557 (fasting insulin) and 42,854 (2hGlu) non-diabetic individuals	MAGIC	European ancestry
Hip and knee OA	Tachmazidou I et al (Tachmazidou et al., 2019)	2019	GWAS of 77,052 cases and 378,169 controls	UKB and arcOGEN Consortium	European ancestry
BMI	Locke AE et al (Locke et al. 2015)	2015	Genome-wide association meta-analysis with 339,224 individuals from 125 studies	GIANT Consortium	European ancestry
Weight	Randall JC et al (Randall et al. 2013)	2013	Genome-wide association meta-analyses of 270,775 individuals from 94 studies	GIANT Consortium	European ancestry
Obesity	Berndt SI et al (Berndt et al. 2013)	2013	Genome-wide association meta-analyses of 263,407 individuals	GIANT Consortium	European ancestry

T2D = type 2 diabetes; FG = fasting glucose; 2hGlu = 2-hour postprandial glucose; OA = osteoarthritis; BMI = Body mass index; GWAS = genome-wide association study; DIAGRAM = DIAbetes Genetics Replication And Meta-analysis; GERA = Genetic Epidemiology Research on Aging; UKB = UK Biobank; MAGIC = Meta-Analyses of Glucose and Insulin-related traits Consortium; arcOGEN = Arthritis Research UK Osteoarthritis Genetics; GIANT=Genetic Investigation of ANthropometric Traits

Table S1. The detailed characteristics of GWAS associated with exposures,outcomes and confounding factors.

Target SNPs	Chr	Mapped genes	Effect allele*	Association with T2D†				Association with hip OA‡			Association with knee OA‡		
				β	SE	*p*-Value	*F* Statistic	β	SE	*p*-Value	β	SE	*p*-Value
rs10114341	9	LOC107987099	C	-0.0409	0.0072	1.15E-08	32.2687	0.0153	0.0117	0.1931	0.0074	0.0094	0.4262
rs1050226	6	SSR1	G	-0.0491	0.0074	3.34E-11	44.0250	-0.0075	0.0118	0.5287	0.0053	0.0094	0.5772
rs1061813	5	ANKH	A	-0.0429	0.0073	3.37E-09	34.5357	0.0123	0.0117	0.2928	-0.0079	0.0093	0.3993
rs10740322	10	RPL5P26; LINC02651	A	0.0477	0.0085	2.11E-08	31.4919	-0.0075	0.0126	0.5540	0.0117	0.0100	0.2423
rs10811661	9	CDKN2B-AS1	C	-0.1569	0.0098	4.13E-58	256.3266	-0.0358	0.0154	0.0201	-0.0015	0.0123	0.9043
rs10842994	12	LOC105369709	T	-0.0755	0.0091	1.02E-16	68.8353	0.0103	0.0146	0.4796	-0.0031	0.0116	0.7922
rs1127655	1	PTGFRN; CD101	T	-0.0438	0.0079	2.47E-08	30.7393	-0.0148	0.0117	0.2048	-0.0081	0.0093	0.3810
rs12617659	2	LOC105373585	T	-0.0685	0.0103	2.83E-11	44.2290	-0.0078	0.0163	0.6348	-0.0130	0.0130	0.3180
rs1333039	9	CDKN2B-AS1	C	0.0534	0.0074	5.64E-13	52.0738	-0.0033	0.0119	0.7790	-0.0083	0.0095	0.3792
rs16988333	22	HORMAD2	G	-0.0745	0.013	9.17E-09	32.8417	-0.0065	0.0201	0.7478	-0.0211	0.0160	0.1856
rs17168486	7	DGKB	T	0.0742	0.0094	2.18E-15	62.3092	-0.0238	0.0154	0.1232	-0.0113	0.0123	0.3600
rs17791483	9	LOC101927450; CHCHD2P9	G	-0.102	0.0147	3.42E-12	48.1466	0.0404	0.0240	0.0920	0.0190	0.0191	0.3204
rs1801214	4	WFS1	T	0.0903	0.0074	5.52E-34	148.9060	0.0029	0.0119	0.8047	0.0104	0.0095	0.2733
rs2191348	7	GTF3AP5; AGMO	T	0.0652	0.0073	3.44E-19	79.7718	-0.0188	0.0117	0.1087	-0.0132	0.0093	0.1572
rs2237892	11	KCNQ1	T	-0.096	0.0157	8.75E-10	37.3889	0.0157	0.0238	0.5099	0.0062	0.0190	0.7436
rs2246618	6	MICB	T	0.0513	0.0084	1.20E-09	37.2972	-0.0055	0.0126	0.6608	0.0153	0.0100	0.1281
rs2261181	12	RPSAP52	T	0.0985	0.0118	9.18E-17	69.6800	0.0138	0.0198	0.4863	-0.0288	0.0158	0.0685
rs2292662	3	SCAANT1; ATXN7	T	-0.0629	0.0111	1.24E-08	32.1111	0.0095	0.0164	0.5608	-0.0095	0.0130	0.4640
rs2294120	8	ZNF34	G	-0.0443	0.0079	1.62E-08	31.4451	0.0087	0.0117	0.4563	-0.0011	0.0093	0.9064
rs2493394	1	NOTCH2	G	0.073	0.0113	1.15E-10	41.7339	-0.0092	0.0188	0.6240	-0.0247	0.0149	0.0978
rs2908282	7	YKT6	A	0.0552	0.0094	4.25E-09	34.4844	-0.0294	0.0153	0.0540	-0.0065	0.0121	0.5946
rs2925979	16	CMIP	C	-0.0534	0.0078	9.06E-12	46.8698	0.0157	0.0127	0.2179	-0.0028	0.0101	0.7832
rs4823182	22	SAMM50	G	0.0482	0.0077	3.36E-10	39.1843	-0.0043	0.0124	0.7298	-0.0143	0.0098	0.1464
rs516946	8	ANK1; MIR486-1	C	0.0824	0.0085	3.16E-22	93.9759	0.0125	0.0137	0.3620	-0.0025	0.0109	0.8160
rs6066138	20	EYA2	A	-0.049	0.0082	1.93E-09	35.7079	-0.0074	0.0130	0.5661	-0.0056	0.0103	0.5856
rs6515236	20	LOC105372562	C	-0.0504	0.0091	3.34E-08	30.6746	-0.0058	0.0135	0.6684	0.0051	0.0107	0.6363
rs7138300	12	TSPAN8; PTPRR	T	-0.0443	0.0072	5.65E-10	37.8567	-0.0108	0.0117	0.3573	-0.0106	0.0094	0.2582
rs7240767	18	LAMA1	C	0.0451	0.0081	2.16E-08	31.0015	1.00E-04	0.0120	0.9930	-0.0055	0.0095	0.5603
rs735949	4	ACSL1	C	-0.0711	0.0106	1.95E-11	44.9912	-0.0042	0.0168	0.8038	-0.0128	0.0134	0.3399
rs7929543	11	LOC729960	C	0.0828	0.0138	2.20E-09	36.0000	-0.0066	0.0211	0.7538	-0.0306	0.0168	0.0681
rs8108269	19	RN7SL836P	G	0.0644	0.0079	3.11E-16	66.4535	0.0088	0.0129	0.4974	-0.0151	0.0103	0.1437
rs840967	2	LOC105374780; LOC105374781	A	-0.0497	0.008	5.44E-10	38.5952	0.0031	0.0119	0.7921	0.0095	0.0095	0.3153
rs982077	15	USP3	G	-0.0453	0.0072	2.58E-10	39.5851	-0.0127	0.0118	0.2793	-0.0015	0.0094	0.8690
rs9844972	3	TSC22D2; LOC107986142	C	0.0956	0.0148	1.03E-10	41.7246	0.0158	0.0229	0.4917	-2.00E-04	0.0184	0.9898
rs993380	4	SCD5	G	-0.0507	0.0081	4.59E-10	39.1783	-0.0125	0.0124	0.3102	-0.0084	0.0098	0.3905

SNP = single nucleotide polymorphism; Chr = chromosome; OA = osteoarthritis; T2D = type 2 diabetes; SE= standard error (the standard error is an estimate of the standard deviation (SD) of the coefficient)

*Allele asociated with higher risk of T2D.

† Effect size estimates for T2D were from DIAbetes Genetics Replication and Meta-analysis (DIAGRAM) Consortium

‡ Effect size estimates for hip and knee OA were from Arthritis Research UK Osteoarthritis Genetics (arcOGEN) Consortium.

#Increase in log-odds of OA (expressed as standard deviations of hip and knee osteoarthritis) per additional exposure-increasing allele.

Table S2. Characteristics of SNPs used in Mendelian randomization analysis of the effects of T2D on hip and knee OA risk.

Exposure	SNP	Chr	Mapped genes	Effect allele*	Confounding factors	*p*-Value
**T2D**	rs10114341	9	LOC107987099	C	**BMI**	0.9698
**T2D**	rs1050226	6	SSR1	G	**BMI**	0.9379
**T2D**	rs1061813	5	ANKH	A	**BMI**	0.6502
**T2D**	rs10740322	10	RPL5P26; LINC02651	A	**BMI**	0.8644
**T2D**	rs10811661	9	CDKN2B-AS1	C	**BMI**	0.3024
**T2D**	rs10842994	12	LOC105369709	T	**BMI**	0.7648
**T2D**	rs1127655	1	PTGFRN; CD101	T	**BMI**	0.4612
**T2D**	rs12617659	2	LOC105373585	T	**BMI**	0.7341
**T2D**	rs1333039	9	CDKN2B-AS1	C	**BMI**	0.9281
**T2D**	rs16988333	22	HORMAD2	G	**BMI**	0.2206
**T2D**	rs17168486	7	DGKB	T	**BMI**	0.2567
**T2D**	rs17791483	9	LOC101927450; CHCHD2P9	G	**BMI**	0.6505
**T2D**	rs1801214	4	WFS1	T	**BMI**	0.2479
**T2D**	rs2191348	7	GTF3AP5; AGMO	T	**BMI**	0.1743
**T2D**	rs2237892	11	KCNQ1	T	**BMI**	0.6165
**T2D**	rs2246618	6	MICB	T	**BMI**	0.5401
**T2D**	rs2261181	12	RPSAP52	T	**BMI**	0.5694
**T2D**	rs2292662	3	SCAANT1; ATXN7	T	**BMI**	0.4562
**T2D**	rs2294120	8	ZNF34	G	**BMI**	0.6601
**T2D**	rs2493394	1	NOTCH2	G	**BMI**	0.8288
**T2D**	rs2908282	7	YKT6	A	**BMI**	0.8360
**T2D**	rs2925979	16	CMIP	C	**BMI**	0.7210
**T2D**	rs4823182	22	SAMM50	G	**BMI**	0.2808
**T2D**	rs516946	8	ANK1; MIR486-1	C	**BMI**	0.4727
**T2D**	rs6066138	20	EYA2	A	**BMI**	0.8818
**T2D**	rs6515236	20	LOC105372562	C	**BMI**	0.9814
**T2D**	rs7138300	12	TSPAN8; PTPRR	T	**BMI**	0.9651
**T2D**	rs7240767	18	LAMA1	C	**BMI**	0.4094
**T2D**	rs735949	4	ACSL1	C	**BMI**	0.1463
**T2D**	rs7929543	11	LOC729960	C	**BMI**	0.5506
**T2D**	rs8108269	19	RN7SL836P	G	**BMI**	0.7244
**T2D**	rs840967	2	LOC105374780; LOC105374781	A	**BMI**	0.8745
**T2D**	rs982077	15	USP3	G	**BMI**	0.3458
**T2D**	rs9844972	3	TSC22D2; LOC107986142	C	**BMI**	0.5078
**T2D**	rs993380	4	SCD5	G	**BMI**	0.6546
**T2D**	rs10114341	9	LOC107987099	C	**Male-weight**	0.9000
**T2D**	rs1050226	6	SSR1	G	**Male-weight**	0.1900
**T2D**	rs1061813	5	ANKH	A	**Male-weight**	0.4500

**T2D**	rs10740322	10	RPL5P26; LINC02651	A	**Male-weight**	0.4900
**T2D**	rs10811661	9	CDKN2B-AS1	C	**Male-weight**	0.8200
**T2D**	rs10842994	12	LOC105369709	T	**Male-weight**	0.4300
**T2D**	rs1127655	1	PTGFRN; CD101	T	**Male-weight**	0.4000
**T2D**	rs12617659	2	LOC105373585	T	**Male-weight**	0.4600
**T2D**	rs1333039	9	CDKN2B-AS1	C	**Male-weight**	0.4000
**T2D**	rs16988333	22	HORMAD2	G	**Male-weight**	0.1600
**T2D**	rs17168486	7	DGKB	T	**Male-weight**	0.8500
**T2D**	rs17791483	9	LOC101927450; CHCHD2P9	G	**Male-weight**	0.5600
**T2D**	rs1801214	4	WFS1	T	**Male-weight**	0.1100
**T2D**	rs2191348	7	GTF3AP5; AGMO	T	**Male-weight**	0.9500
**T2D**	rs2237892	11	KCNQ1	T	**Male-weight**	0.2900
**T2D**	rs2246618	6	MICB	T	**Male-weight**	0.6600
**T2D**	rs2261181	12	RPSAP52	T	**Male-weight**	0.1600
**T2D**	rs2292662	3	SCAANT1; ATXN7	T	**Male-weight**	0.1400
**T2D**	rs2294120	8	ZNF34	G	**Male-weight**	0.2200
**T2D**	rs2493394	1	NOTCH2	G	**Male-weight**	0.3800
**T2D**	rs2908282	7	YKT6	A	**Male-weight**	0.2400
**T2D**	rs2925979	16	CMIP	C	**Male-weight**	0.2700
**T2D**	rs4823182	22	SAMM50	G	**Male-weight**	0.5300
**T2D**	rs516946	8	ANK1; MIR486-1	C	**Male-weight**	0.6000
**T2D**	rs6066138	20	EYA2	A	**Male-weight**	0.8200
**T2D**	rs6515236	20	LOC105372562	C	**Male-weight**	0.4600
**T2D**	rs7138300	12	TSPAN8; PTPRR	T	**Male-weight**	0.6300
**T2D**	rs7240767	18	LAMA1	C	**Male-weight**	0.4800
**T2D**	rs735949	4	ACSL1	C	**Male-weight**	0.3400
**T2D**	rs7929543	11	LOC729960	C	**Male-weight**	0.8100
**T2D**	rs8108269	19	RN7SL836P	G	**Male-weight**	0.6600
**T2D**	rs840967	2	LOC105374780; LOC105374781	A	**Male-weight**	0.7200
**T2D**	rs982077	15	USP3	G	**Male-weight**	0.4600
**T2D**	rs9844972	3	TSC22D2; LOC107986142	C	**Male-weight**	0.3800
**T2D**	rs993380	4	SCD5	G	**Male-weight**	0.8200
**T2D**	rs10114341	9	LOC107987099	C	**Female-weight**	0.9900
**T2D**	rs1050226	6	SSR1	G	**Female-weight**	0.7000
**T2D**	rs1061813	5	ANKH	A	**Female-weight**	0.9400
**T2D**	rs10740322	10	RPL5P26; LINC02651	A	**Female-weight**	0.1100
**T2D**	rs10811661	9	CDKN2B-AS1	C	**Female-weight**	1.0000
**T2D**	rs10842994	12	LOC105369709	T	**Female-weight**	0.1300
**T2D**	rs1127655	1	PTGFRN; CD101	T	**Female-weight**	0.8400
**T2D**	rs12617659	2	LOC105373585	T	**Female-weight**	0.1200
**T2D**	rs1333039	9	CDKN2B-AS1	C	**Female-weight**	0.8700
**T2D**	rs16988333	22	HORMAD2	G	**Female-weight**	0.5600
**T2D**	rs17168486	7	DGKB	T	**Female-weight**	0.7000
**T2D**	rs17791483	9	LOC101927450; CHCHD2P9	G	**Female-weight**	0.3200

**T2D**	rs1801214	4	WFS1	T	**Female-weight**	0.2300
**T2D**	rs2191348	7	GTF3AP5; AGMO	T	**Female-weight**	0.3900
**T2D**	rs2237892	11	KCNQ1	T	**Female-weight**	0.4800
**T2D**	rs2246618	6	MICB	T	**Female-weight**	0.2400
**T2D**	rs2261181	12	RPSAP52	T	**Female-weight**	0.6100
**T2D**	rs2292662	3	SCAANT1; ATXN7	T	**Female-weight**	0.4100
**T2D**	rs2294120	8	ZNF34	G	**Female-weight**	0.4000
**T2D**	rs2493394	1	NOTCH2	G	**Female-weight**	0.3500
**T2D**	rs2908282	7	YKT6	A	**Female-weight**	0.6000
**T2D**	rs2925979	16	CMIP	C	**Female-weight**	0.1500
**T2D**	rs4823182	22	SAMM50	G	**Female-weight**	0.7500
**T2D**	rs516946	8	ANK1; MIR486-1	C	**Female-weight**	0.8300
**T2D**	rs6066138	20	EYA2	A	**Female-weight**	0.9800
**T2D**	rs6515236	20	LOC105372562	C	**Female-weight**	0.6500
**T2D**	rs7138300	12	TSPAN8; PTPRR	T	**Female-weight**	0.3700
**T2D**	rs7240767	18	LAMA1	C	**Female-weight**	0.4500
**T2D**	rs735949	4	ACSL1	C	**Female-weight**	0.5400
**T2D**	rs7929543	11	LOC729960	C	**Female-weight**	0.1300
**T2D**	rs8108269	19	RN7SL836P	G	**Female-weight**	0.7600
**T2D**	rs840967	2	LOC105374780; LOC105374781	A	**Female-weight**	0.7200
**T2D**	rs982077	15	USP3	G	**Female-weight**	0.4700
**T2D**	rs9844972	3	TSC22D2; LOC107986142	C	**Female-weight**	0.7000
**T2D**	rs993380	4	SCD5	G	**Female-weight**	0.3800
**T2D**	rs10114341	9	LOC107987099	C	**Obesity**	0.6300
**T2D**	rs1050226	6	SSR1	G	**Obesity**	0.9600
**T2D**	rs1061813	5	ANKH	A	**Obesity**	0.8200
**T2D**	rs10740322	10	RPL5P26; LINC02651	A	**Obesity**	0.5100
**T2D**	rs10811661	9	CDKN2B-AS1	C	**Obesity**	0.8700
**T2D**	rs10842994	12	LOC105369709	T	**Obesity**	0.4400
**T2D**	rs1127655	1	PTGFRN; CD101	T	**Obesity**	0.1900
**T2D**	rs12617659	2	LOC105373585	T	**Obesity**	0.6800
**T2D**	rs1333039	9	CDKN2B-AS1	C	**Obesity**	0.6400
**T2D**	rs16988333	22	HORMAD2	G	**Obesity**	0.8300
**T2D**	rs17168486	7	DGKB	T	**Obesity**	0.5700
**T2D**	rs17791483	9	LOC101927450; CHCHD2P9	G	**Obesity**	0.1700
**T2D**	rs1801214	4	WFS1	T	**Obesity**	0.8100
**T2D**	rs2191348	7	GTF3AP5; AGMO	T	**Obesity**	0.5100
**T2D**	rs2237892	11	KCNQ1	T	**Obesity**	0.3300
**T2D**	rs2246618	6	MICB	T	**Obesity**	0.3600
**T2D**	rs2261181	12	RPSAP52	T	**Obesity**	0.2100
**T2D**	rs2292662	3	SCAANT1; ATXN7	T	**Obesity**	0.8500
**T2D**	rs2294120	8	ZNF34	G	**Obesity**	0.4800
**T2D**	rs2493394	1	NOTCH2	G	**Obesity**	0.8500
**T2D**	rs2908282	7	YKT6	A	**Obesity**	0.8400

**T2D**	rs2925979	16	CMIP	C	**Obesity**	0.3400
**T2D**	rs4823182	22	SAMM50	G	**Obesity**	0.1700
**T2D**	rs516946	8	ANK1; MIR486-1	C	**Obesity**	0.8900
**T2D**	rs6066138	20	EYA2	A	**Obesity**	0.4200
**T2D**	rs6515236	20	LOC105372562	C	**Obesity**	0.6600
**T2D**	rs7138300	12	TSPAN8; PTPRR	T	**Obesity**	0.8400
**T2D**	rs7240767	18	LAMA1	C	**Obesity**	0.2500
**T2D**	rs735949	4	ACSL1	C	**Obesity**	0.4100
**T2D**	rs7929543	11	LOC729960	C	**Obesity**	0.7500
**T2D**	rs8108269	19	RN7SL836P	G	**Obesity**	0.8400
**T2D**	rs840967	2	LOC105374780; LOC105374781	A	**Obesity**	0.4800
**T2D**	rs982077	15	USP3	G	**Obesity**	0.5900
**T2D**	rs9844972	3	TSC22D2; LOC107986142	C	**Obesity**	0.1300
**T2D**	rs993380	4	SCD5	G	**Obesity**	0.7900

T2D = type 2 diabetes; SNP = single nucleotide polymorphism; Chr = chromosome; BMI = body mass index

*Allele asociated with higher risk of T2D.

Table S3. Associations of SNPs used in Mendelian randomization analysis of T2D and osteoarthritis risk with confounding factors.

Target SNPs	Chr	Mapped genes	Effect allele*	Association with FG†				Association with hip OA‡			Association with knee OA‡		
				β	SE	*p*-Value	*F* statistic	β#	SE	*p*-Value	β#	SE	*p*-Value
rs11195502	10	BTBD7P2	C	0.0320	0.0037	1.97E-18	74.7991	-0.0158	0.0203	0.4351	-0.0312	0.0162	0.0534
rs11607883	11	SLC35C1; CRY2	G	0.0210	0.0021	6.32E-24	100.0000	0.0226	0.0117	0.0527	-0.0153	0.0093	0.0992
rs16913693	9	ELP1	T	0.0430	0.0066	3.51E-11	42.4472	-0.0577	0.0369	0.1178	-0.0411	0.0296	0.1660
rs17168486	7	DGKB	C	-0.0310	0.0028	3.17E-28	122.5765	-0.0238	0.0154	0.1232	-0.0113	0.0123	0.3600
rs174576	11	WARS	C	0.0200	0.0022	1.18E-18	82.6446	0.0056	0.0122	0.6468	-0.0067	0.0097	0.4933
rs2191349	7	GTF3AP5; DGKB	G	-0.0290	0.0021	1.28E-42	190.7029	-0.0188	0.0117	0.1082	-0.0122	0.0093	0.1922
rs4502156	15	NPM1P47	T	0.0220	0.0021	1.38E-25	109.7506	-0.0020	0.0117	0.8642	-0.0129	0.0093	0.1678
rs6072275	20	PLCG1-AS1; TOP1	G	-0.0160	0.0028	1.66E-08	32.6531	0.0088	0.0162	0.5872	-0.0043	0.0129	0.7383
rs6975024	7	GCK, YKT6	T	-0.0610	0.0029	2.88E-99	442.4495	0.0291	0.0153	0.0564	0.0078	0.0121	0.5184
rs882020	7	MYL7	C	-0.0210	0.0030	3.04E-12	49.0000	0.0084	0.0174	0.6288	0.0256	0.0138	0.0633

SNP = single nucleotide polymorphism; Chr = chromosome; FG = fasting glucose; SE= standard error (the standard error is an estimate of the standard deviation (SD) of the coefficient)

*Allele asociated with higher level of fasting glucose in nondiabetic patients.

† Effect size estimates for fasting glucose were from The Meta-Analyses of Glucose and Insulin-related traits Consortium (MAGIC).

‡ Effect size estimates for hip and knee osteoarthritis were from Arthritis Research UK Osteoarthritis Genetics (arcOGEN) Consortium.

# Increase in log-odds of osteoarthritis (expressed as standard deviations of hip and knee osteoarthritis) per additional exposure-increasing allele.

Table S4. Characteristics of SNPs used in Mendelian randomization analysis of the effects of FG in nondiabetic individuals on hip and knee OA risk.

Exposure	SNP	Chr	Mapped genes	Effect allele*	Confounding factors	*p*-Value
**FG**	rs11195502	10	BTBD7P2	C	**BMI**	0.5028
**FG**	rs11607883	11	SLC35C1; CRY2	G	**BMI**	0.9714
**FG**	rs16913693	9	ELP1	T	**BMI**	0.4942
**FG**	rs17168486	7	DGKB	C	**BMI**	0.2567
**FG**	rs174576	11	WARS	C	**BMI**	0.4028
**FG**	rs2191349	7	GTF3AP5; DGKB	G	**BMI**	0.1959
**FG**	rs4502156	15	NPM1P47	T	**BMI**	0.1387
**FG**	rs6072275	20	PLCG1-AS1; TOP1	G	**BMI**	0.7243
**FG**	rs6975024	7	GCK, YKT6	T	**BMI**	0.8994
**FG**	rs882020	7	MYL7	C	**BMI**	0.6751
**FG**	rs11195502	10	BTBD7P2	C	**Male-weight**	0.6100
**FG**	rs11607883	11	SLC35C1; CRY2	G	**Male-weight**	0.4500
**FG**	rs16913693	9	ELP1	T	**Male-weight**	0.1800
**FG**	rs17168486	7	DGKB	C	**Male-weight**	0.8500
**FG**	rs174576	11	WARS	C	**Male-weight**	0.4600
**FG**	rs2191349	7	GTF3AP5; DGKB	G	**Male-weight**	0.9600
**FG**	rs4502156	15	NPM1P47	T	**Male-weight**	0.6600
**FG**	rs6072275	20	PLCG1-AS1; TOP1	G	**Male-weight**	0.3200
**FG**	rs6975024	7	GCK, YKT6	T	**Male-weight**	0.1800
**FG**	rs882020	7	MYL7	C	**Male-weight**	0.7400
**FG**	rs11195502	10	BTBD7P2	C	**Female-weight**	0.1600
**FG**	rs11607883	11	SLC35C1; CRY2	G	**Female-weight**	0.4800
**FG**	rs16913693	9	ELP1	T	**Female-weight**	0.8900
**FG**	rs17168486	7	DGKB	C	**Female-weight**	0.7000
**FG**	rs174576	11	WARS	C	**Female-weight**	0.9100
**FG**	rs2191349	7	GTF3AP5; DGKB	G	**Female-weight**	0.3800
**FG**	rs4502156	15	NPM1P47	T	**Female-weight**	0.3700
**FG**	rs6072275	20	PLCG1-AS1; TOP1	G	**Female-weight**	0.6100
**FG**	rs6975024	7	GCK, YKT6	T	**Female-weight**	0.6000
**FG**	rs882020	7	MYL7	C	**Female-weight**	0.1600
**FG**	rs11195502	10	BTBD7P2	C	**Obesity**	0.1200
**FG**	rs11607883	11	SLC35C1; CRY2	G	**Obesity**	0.4300
**FG**	rs16913693	9	ELP1	T	**Obesity**	0.9600
**FG**	rs17168486	7	DGKB	C	**Obesity**	0.5700
**FG**	rs174576	11	WARS	C	**Obesity**	0.3400
**FG**	rs2191349	7	GTF3AP5; DGKB	G	**Obesity**	0.5200
**FG**	rs4502156	15	NPM1P47	T	**Obesity**	0.6000
**FG**	rs6072275	20	PLCG1-AS1; TOP1	G	**Obesity**	0.5600
**FG**	rs6975024	7	GCK, YKT6	T	**Obesity**	0.8100
**FG**	rs882020	7	MYL7	C	**Obesity**	0.4500

SNP = single nucleotide polymorphism; Chr = chromosome; FG = fasting glucose; BMI = body mass index

*Allele asociated with higher level of FG.

Table S5. Associations of SNPs used in Mendelian randomization analysis of FG and osteoarthritis risk with confounding factors.

Target SNPs	Chr	Mapped genes	Effect allele*	Association with 2hGlu†				Association with hip OA‡			Association with knee OA‡		
				β	SE	*p*-Value	*F* statistic	β	SE	*p*-Value	β	SE	*p*-Value
rs1019503	5	ERAP1; ERAP2	A	0.0630	0.0110	8.87E-09	32.8017	0.0179	0.0117	0.1249	-0.0024	0.0093	0.7936
rs6547829	2	BABAM2	T	0.1100	0.0190	3.13E-08	33.5180	-0.0103	0.0207	0.6181	0.0138	0.0165	0.4011
rs6975024	7	GCK; YKT6	C	-0.1000	0.0160	5.25E-11	39.0625	-0.0291	0.0153	0.0564	-0.0078	0.0121	0.5184

SNP = single nucleotide polymorphism; Chr = chromosome; 2hGlu = 2-hour postprandial glucose; SE= standard error (the standard error is an estimate of the standard deviation (SD) of the coefficient)

*Allele asociated with higher level of 2-hour postprandial glucose in nondiabetic patients.

†Effect size estimates for 2-hour postprandial glucose were from The Meta-Analyses of Glucose and Insulin-related traits Consortium (MAGIC).

‡Effect size estimates for hip and knee osteoarthritis were from Arthritis Research UK Osteoarthritis Genetics (arcOGEN) Consortium.

# Increase in log-odds of osteoarthritis (expressed as standard deviations of hip and knee osteoarthritis) per additional exposure-increasing allele.

Table S6. Characteristics of SNPs used in Mendelian randomization analysis of the effects of 2hGlu in nondiabetic individuals on hip and knee OA risk.

Exposure	SNP	Chr	Mapped genes	Effect allele[*]	Confounding factors	*p*-Value
**2hGlu**	rs1019503	5	ERAP1; ERAP2	A	**BMI**	0.7476
**2hGlu**	rs6547829	2	BABAM2	T	**BMI**	0.3930
**2hGlu**	rs6975024	7	GCK; YKT6	C	**BMI**	0.8994
**2hGlu**	rs1019503	5	ERAP1; ERAP2	A	**Male-weight**	0.6200
**2hGlu**	rs6547829	2	BABAM2	T	**Male-weight**	0.8600
**2hGlu**	rs6975024	7	GCK; YKT6	C	**Male-weight**	0.1800
**2hGlu**	rs1019503	5	ERAP1; ERAP2	A	**Female-weight**	0.5600
**2hGlu**	rs6547829	2	BABAM2	T	**Female-weight**	0.7500
**2hGlu**	rs6975024	7	GCK; YKT6	C	**Female-weight**	0.6000
**2hGlu**	rs1019503	5	ERAP1; ERAP2	A	**Obesity**	0.9500
**2hGlu**	rs6547829	2	BABAM2	T	**Obesity**	0.5200
**2hGlu**	rs6975024	7	GCK; YKT6	C	**Obesity**	0.8100

SNP = single nucleotide polymorphism; Chr = chromosome; 2hGlu = 2-hour postprandial glucose; BMI = body mass index

*Allele asociated with higher level of 2hGlu.

Table S7. Associations of SNPs used in Mendelian randomization analysis of 2hGlu and osteoarthritis risk with confounding factors.
